# Ammonia promotes the proliferation of bone marrow-derived mesenchymal stem cells by regulating the Akt/mTOR/S6k pathway

**DOI:** 10.1038/s41413-022-00215-y

**Published:** 2022-08-26

**Authors:** Yu Liu, Xiangxian Zhang, Wei Wang, Ting Liu, Jun Ren, Siyuan Chen, Tianqi Lu, Yan Tie, Xia Yuan, Fei Mo, Jingyun Yang, Yuquan Wei, Xiawei Wei

**Affiliations:** 1 Laboratory of Aging Research and Cancer Drug Target, State Key Laboratory of Biotherapy and Cancer Center, National Clinical Research Center for Geriatrics, West China Hospital, Sichuan University, No. 17, Block 3, Southern Renmin Road, Chengdu, Sichuan 610041 PR China; 2 Department of Clinical Laboratory, The West China Second University Hospital of Sichuan University (WCSUH-SCU), Sichuan University, No. 17, Block 3, Southern Renmin Road, Chengdu, Sichuan 610041 PR China; 3 Department of Prenatal Diagnosis Center, The West China Second University Hospital of Sichuan University (WCSUH-SCU), Sichuan University, No. 17, Block 3, Southern Renmin Road, Chengdu, Sichuan 610041 PR China

**Keywords:** Bone cancer, Bone

## Abstract

Ammonia plays an important role in cellular metabolism. However, ammonia is considered a toxic product. In bone marrow-derived mesenchymal stem cells, multipotent stem cells with high expression of glutamine synthetase (GS) in bone marrow, ammonia and glutamate can be converted to glutamine via glutamine synthetase activity to support the proliferation of MSCs. As a major nutritional amino acid for biosynthesis, glutamine can activate the Akt/mTOR/S6k pathway to stimulate cell proliferation. The activation of mTOR can promote cell entry into S phase, thereby enhancing DNA synthesis and cell proliferation. Our studies demonstrated that mesenchymal stem cells can convert the toxic waste product ammonia into nutritional glutamine via GS activity. Then, the Akt/mTOR/S6k pathway is activated to promote bone marrow-derived mesenchymal stem cell proliferation. These results suggest a new therapeutic strategy and potential target for the treatment of diseases involving hyperammonemia.

## Introduction

Ammonia is a main product of many cellular metabolic pathways.^1^ Physiologically, the normal ammonia concentration ranges from 20 to 50 μmol·L^−1^ in the blood of healthy humans. Ammonia concentrations can exceed normal levels, such as in uremia, a terminal stage of chronic renal failure that is characterized by high ammonia concentrations, or in tumor patients, who showed higher ammonia concentrations ranging from 2 to 5 mmol·L^−1^.^2^ Excess ammonia is considered a toxic product and is transported to the liver for entry into the urea cycle and then excreted through the kidney in the form of urea. However, when the ammonia concentration exceeds the maximum level that can be cleared liver metabolism, the accumulation of excessive ammonia results in hyperammonaemia.^3^ The increased level of ammonia can suppress the majority of cell proliferation and cause cell apoptosis or necrosis due to the toxicity of ammonia.^4^

As noted above, the urea cycle of the liver is the main ammonia metabolic pathway.^5^ In addition, ammonia can be converted to amino acids or other nitrogenous compounds to maintain the nitrogen balance; the most vital nutritional amino acid is glutamine.^6^ Glutamine is an extremely important nutritional amino acid. As a supplement of various metabolic pathways, glutamine plays a prominent role in cell proliferation.^7^ Glutamine is not only a precursor for nucleotide synthesis^8^ but can also activate the Akt/mTOR/S6k pathways by increasing the phosphorylation of mTOR (Ser2448), which is a common nutritional regulatory pathway protein.^9^ Mammalian target of rapamycin (mTOR), a common serine/threonine protein kinase, is a part of the phosphatidylinositol 3-kinase-related kinase family.^10^ mTOR is a key regulator of cell proliferation that is modulated by growth factors, nutrition and amino acids.^11^ Moreover, mTOR is also involved in regulating the cell cycle,^12^ and a key event is modulating cells from G1 phase to S phase through the activation of cyclin D/CDK4 and cyclin E/CDK2, which then activate the E2F transcription system to promote cell entry into S phase.^13^ Cells in S phase increase DNA synthesis and actively proliferate.^14^

Mesenchymal stem cells (MSCs), also called multipotent mesenchymal stromal cells,^15^ have the ability to self-renew and differentiate into various types of cells, such as osteocytes, adipocytes, and chondrocytes.^16^ Our study detected high expression of glutamine synthetase (GS) in MSCs, indicating ammonia metabolism in the MSCs. Glutamine synthetase is a key enzyme that utilizes glutamate and ammonia to synthesize glutamine. Glutamine synthetase is a significant metabolic pathway for the transport of excess ammonia in the body.^17^ As previously noted, excess ammonia is toxic to organisms; however, with high expression of glutamine synthetase, ammonia is no longer a toxic product in MSCs, and it can be converted to the nutritional amino acid glutamine via glutamine synthetase (GS) activity.^18^ In this report, we revealed that excessive ammonia is no longer a toxic product but a nutritional molecule for MSCs through catalysis of GS and activation of the mTOR pathway.

## Results

### Isolation and identification of bone marrow-derived mesenchymal stem cells from mouse bone marrow

**Fig. 1:**
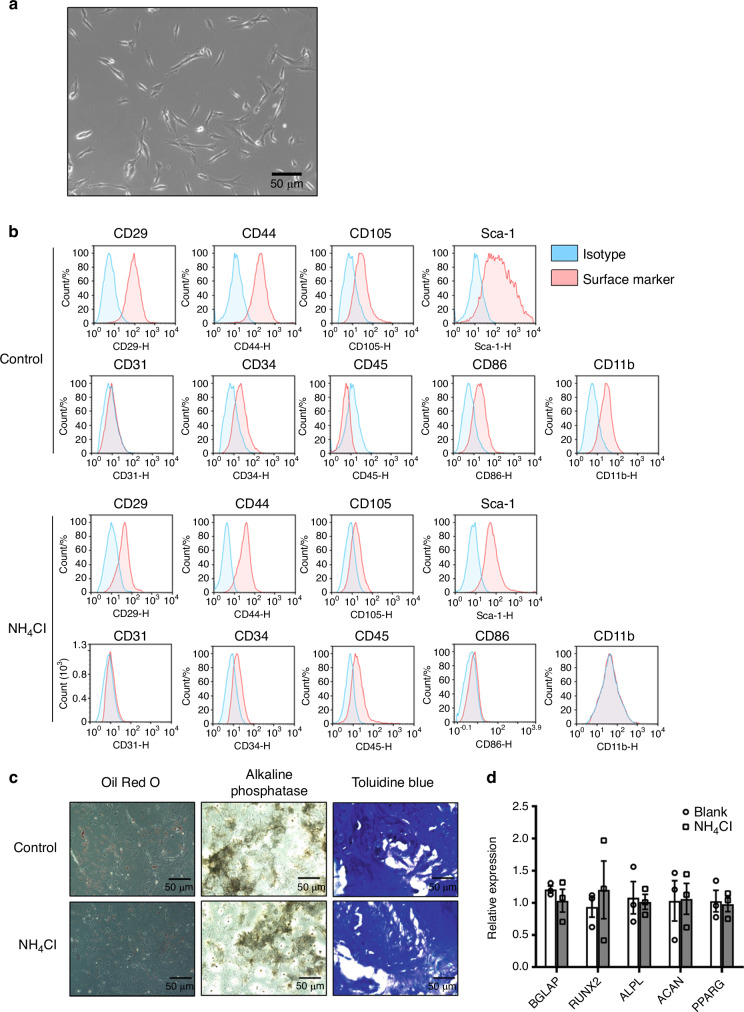
Isolation and identification of mesenchymal stem cells from mouse bone marrow. **a** Fibroblast-like or spindle-shaped morphology of mesenchymal stem cells appears at passages 4–6. **b** Passage 4–6 cells were harvested and stained with positive surface markers of mouse BM-MSCs: CD29, CD44, CD105, and Sca-1 and negative markers: CD31, CD34, CD45, CD86, and CD11b with their corresponding isotype control via FACS analysis. **c** Adipogenesis of MSCs was observed with oil red O staining, osteoblastogenesis was assayed with in situ alkaline phosphatase staining, and chondrocytic cells were identified with toluidine blue staining. **d** RT-PCR results showing the relative expression of *bglap, runx2, alpl, acan* and *pparg* with or without NH_4_Cl treatment

MSCs were isolated from the femurs and tibias of 6-week-old C57 mice.^19^ After 4–6 passages, MSCs demonstrated a fibroblast-like or spindle-like shape in culture (Fig. 1a). Then, we identified the surface markers of MSCs with or without ammonia treatment via flow cytometry analysis,^20^ including the positive markers of MSCs (CD44, Sca-1, CD29, CD105) and the negative markers of MSCs (CD31, CD86, CD11b, CD34, CD45) (Fig. 1b). To evaluate the multidifferentiation capacity of MSCs, we used a differentiation assays in vitro by differentiating these cells into different lineages, including osteoblasts, adipocytes and chondrocytes. Then, we verified osteogenic induction with positive alkaline phosphatase staining, adipogenic induction with positive oil red O staining and chondrogenic induction with positive toluidine blue O staining (Fig. 1c). To confirm that ammonia could not affect the characteristics of mesenchymal stem cells, we also detected MSC-related genes, including *Bglap, Runx2, Alpl, Acan* and *Pparg*.^16^ After treatment with or without ammonia, we found no significant difference between the two groups (Fig. 1d), demonstrating that ammonia could not change the characteristics of mesenchymal stem cells.

### Ammonia promotes bone marrow-derived mesenchymal stem cell proliferation

**Fig. 2:**
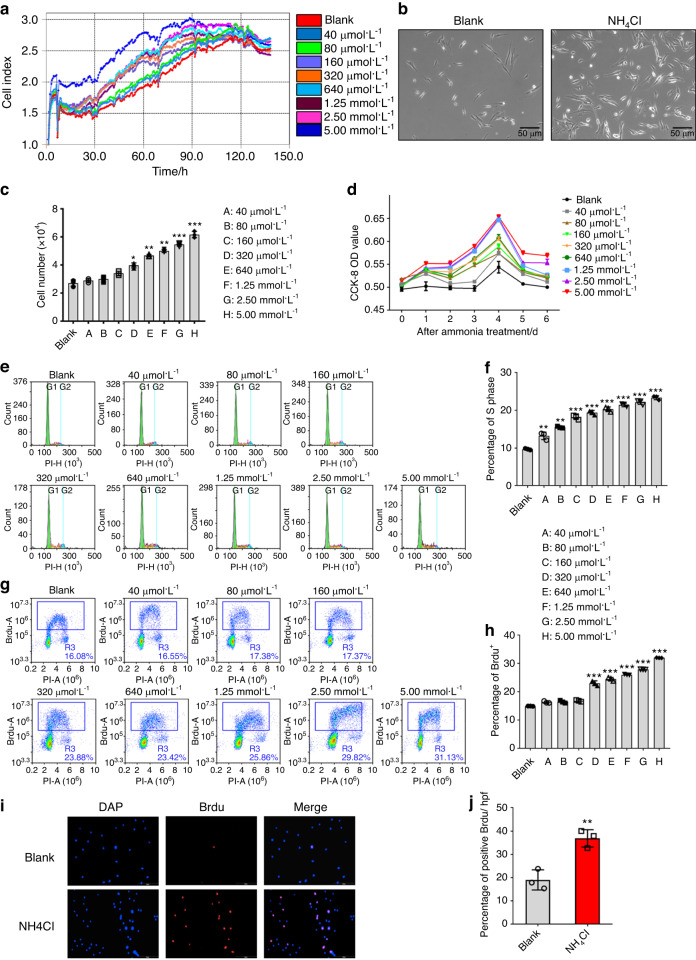
Ammonia promotes mesenchymal stem cell proliferation. **a** RTCA (real-time cellular analysis) of different concentrations of NH_4_Cl (0, 40 µmol·L^−1^, 80 µmol·L^−1^, 160 µmol·L^−1^, 320 µmol·L^−1^, 640 µmol·L^−1^, 1.25 mmol·L^−1^, 2.50 mmol·L^−1^, 5.00 mmol·L^−1^, and 10 mmol·L^−1^). **b** The growth of MSCs with or without NH_4_Cl treatment under a microscope after 48 h. **c** Cell counts after treatment with different concentrations of NH_4_Cl (0, 40 µmol·L^−1^, 80 µmol·L^−1^, 160 µmol·L^−1^, 320 µmol·L^−1^, 640 µmol·L^−1^, 1.25 mmol·L^−1^, 2.50 mmol·L^−1^, 5.00 mmol·L^−1^, and 10 mmol·L^−1^) after 48 h. **d** CCK-8 analysis with different concentrations of NH_4_Cl treatment (0, 40 µmol·L^−1^, 80 µmol·L^−1^, 160 µmol·L^−1^, 320 µmol·L^−1^, 640 µmol·L^−1^, 1.25 mmol·L^−1^, 2.50 mmol·L^−1^, 5.00 mmol·L^−1^, and 10 mmol·L^−1^) lasting 1 week (right). **e** PI flow cytometry analysis. **f** The statistical results of the percentage of S phase cells. **f** BrdU flow cytometry analysis. **g** The statistical results of the percentage of BrdU^+^ cells. **h** Immunofluorescence analysis of BrdU. **j** The statistical results of BrdU-positive cells/HPF. Values are the mean ± SEM of an experiment performed in triplicate (a two-tailed, paired Student’s *t* test). *P < 0.05 versus the controls. ^#^*P* < 0.05 versus the 5 mmol·L^−1^ NH_4_Cl treatment group

To assess the effect of ammonia on MSC proliferation, we added NH_4_Cl in culture with different concentrations of ammonia (0 µmol·L^−1^, 40 µmol·L^−1^, 80 µmol·L^−1^, 160 µmol·L^−1^, 320 µmol·L^−1^, 640 µmol·L^−1^, 1.25 mmol·L^−1^, 2.5 mmol·L^−1^ and 5 mmol·L^−1^), which included the physiological ammonia concentration and an ammonia concentration as high as that in tumors.^21^ Then, we observed the proliferation of mesenchymal stem cells within a week after NH_4_Cl treatment via RTCA (Real Time Cellular Analysis). The results revealed that the NH_4_Cl treatment group, especially the 5 mmol·L^−1^ group, showed a higher cell index than the other groups. After a 96 h NH_4_Cl treatment, there was a downward trend in all growth curves (Fig. 2a). Moreover, there were more MSCs in the NH_4_Cl treatment group than in the non-NH_4_Cl treatment group under the microscope (Fig. 2b), especially in the 5 mmol·L^−1^ ammonia treatment group. When the ammonia concentration exceeded 5 mmol·L^−1^, the effect of ammonia-induced proliferation of MSCs was reduced. These data demonstrated that 5 mmol·L^−1^ NH_4_Cl is the optimal concentration for the proliferation of MSCs. The cell number count of MSCs showed the same result (Fig. 2c). We measured cell proliferation by a 7-day CCK-8 assay, which showed that the ammonia treatment group increased cell proliferation compared to the control group; moreover, the most significant effect on proliferation was on the 2nd day (Fig. 2d). Then, we detected the cell cycle distribution of MSCs with or without 48 h ammonia treatment via PI flow cytometry analysis.^22^ The results demonstrated that the percentage of S-phase cells increased in the NH_4_Cl treatment group compared with the control group (Fig. 2e, f). We also assessed cell proliferation by BrdU incorporation with or without 48 h of treatment with NH_4_Cl^23^ and then determined the percentage of BrdU-positive cells by flow cytometry analysis and immunofluorescence analysis. The results showed that there was a greater percentage of BrdU-positive cells in the NH_4_Cl treatment group than in the control group both in the flow cytometry analysis (Fig. 2g, h) and immunofluorescence analysis (Fig. 2i, j). These results indicated that ammonia at a certain concentration could promote mesenchymal stem cell proliferation.

### Glutamine promotes bone marrow-derived mesenchymal stem cell proliferation

**Fig. 3:**
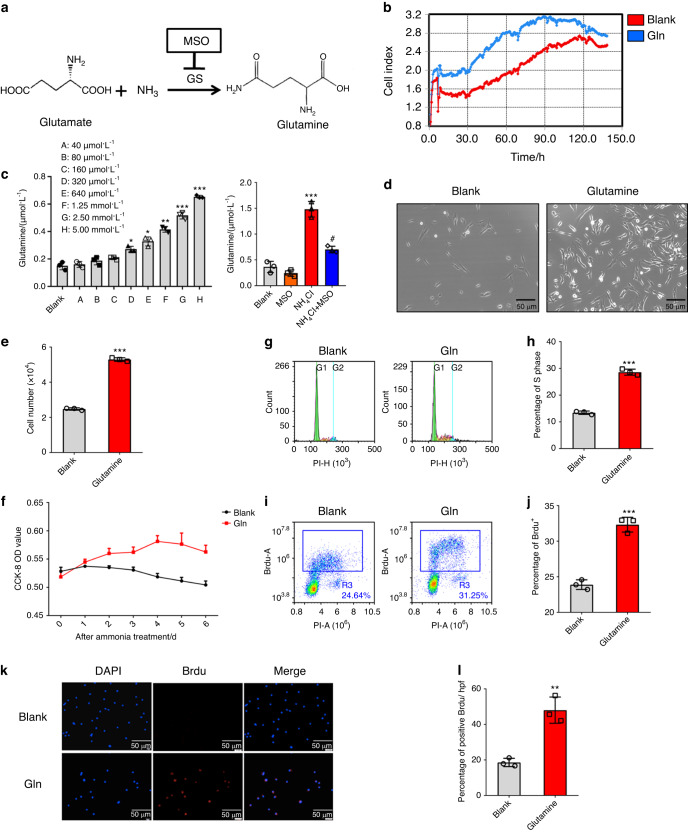
Glutamine promotes mesenchymal stem cell proliferation. **a** Metabolic pathway of ammonia in GS-expressing MSCs. **b** RTCA (real-time cellular analysis) with or without glutamine treatment. **c** Determination of glutamine after NH_4_Cl treatment (left) and with or without both NH_4_Cl treatment and MSO treatment (right). **d** The growth of MSCs with or without glutamine treatment under a microscope. **e** The results of cell count analysis with or without glutamine treatment. **f** CCK-8 analysis with or without glutamine treatment lasting 1 week. **g** PI flow analysis. **h** The statistical results of the percentage of S phase cells. **i** BrdU flow analysis. **j** The statistical results of the percentage of BrdU^+^ cells. **k** Immunofluorescence analysis of BrdU. **l** The statistical results of BrdU^+^ cells/HPF. Values are the mean ± SEM of an experiment performed in triplicate (one-way analysis of variance). *P < 0.05 versus the controls. ^#^*P* < 0.05 versus the 5 mmol·L^−1^ NH_4_Cl treatment group

To investigate the mechanisms of the proliferation of MSCs by ammonia, as a nitrogen supplement of amino acids, we considered that ammonia might be converted with glutamate into glutamine via GS (Fig. 3a). Previously, glutamine was shown to play a prominent role in cell growth and proliferation.^24^ Additionally, more glutamine production was observed in the groups with NH_4_Cl treatment than in the control group (Fig. 3b), and after treatment with MSO, a specific inhibitor of glutamine synthetase,^18^ we found that combined treatment of NH_4_Cl and MSO resulted in decreased production of glutamine compared to single treatment of NH_4_Cl (Fig. 3b). We verified the effect of glutamine on MSC proliferation with 5 mmol·L^−1^ glutamine treatment within a week in vitro via RTCA (Real Time Cellular Analysis). The growth curve of MSCs showed a higher cell index in the glutamine-treated group than in the control group (Fig. 3c). Additionally, there were more MSCs in the glutamine treatment group than in the control group under the microscope (Fig. 3d), and the results of the cell counting analysis demonstrated that the number of glutamine-treated cells was obviously higher than that in the DMEM group (Fig. 3e). The results revealed increased cell proliferation in the glutamine treatment group compared to the control group by 7-day CCK-8 analysis, and the most significant effect on proliferation was observed on the 2nd day (Fig. 3f). Furthermore, the percentage of S phase cells increased in the glutamine treatment group compared to the control group, as shown by PI flow cytometry analysis (Fig. 3g, h), and the percentage of BrdU-positive cells was detected via both immunofluorescence analysis and flow cytometry analysis. The results demonstrated that an increase in BrdU-positive cells was observed in the glutamine treatment group (Fig. 3i–l). These results not only indicated that glutamine could promote MSC proliferation but also indicated that the effects of ammonia on MSC proliferation might be due to the conversion of ammonia to glutamine.

### Inhibition of glutamine synthetase reduces the ammonia-induced proliferation of bone marrow-derived mesenchymal stem cells

**Fig. 4:**
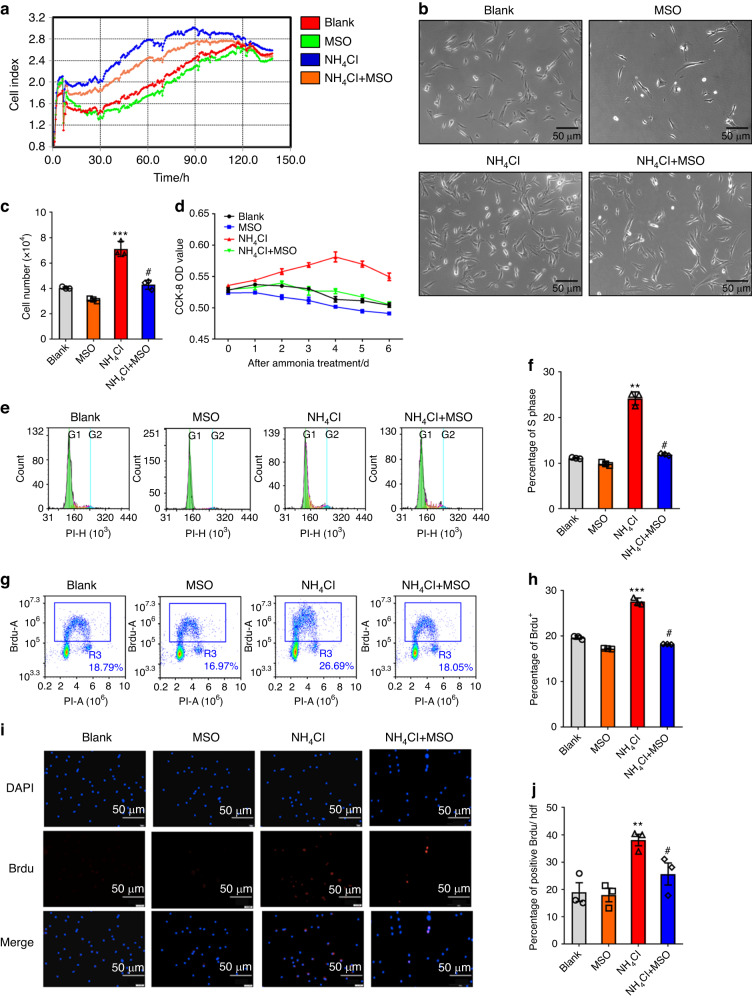
Inhibition of glutamine synthetase reduces the ammonia-induced proliferation of mesenchymal stem cells. **a** RTCA (real-time cellular analysis) with or without both NH_4_Cl treatment or MSO treatment. **b** The growth of MSCs with or without both NH_4_Cl treatment and MSO treatment under a microscope. **c** The cell count results with or without both NH_4_Cl treatment and MSO treatment. **d** CCK-8 analysis with or without NH_4_Cl treatment or MSO treatment after 48 h (left) and lasting 1 week (right). **e** PI flow analysis. **f** The statistical results of the percentage of S phase cells. **g** BrdU flow analysis. **h** The statistical results of the percentage of BrdU^+^ cells. **i** Immunofluorescence analysis of BrdU. **j** The statistical results of BrdU^+^ cells/HPF. Values are the mean ± SEM of an experiment performed in triplicate (one-way analysis of variance). *P < 0.05 versus the controls. ^#^*P* < 0.05 versus the 5 mmol·L^−1^ NH_4_Cl treatment group

To confirm our hypothesis that ammonia could be converted into glutamine to promote MSC proliferation, we used MSO, a specific inhibitor of GS, to determine whether the proliferative effect of ammonia on MSCs could be reduced after inhibition of GS activity. To verify the effect of MSO on MSC proliferation induced by ammonia, we performed RTCA (Real Time Cellular Analysis) within a week. The results demonstrated that after MSO treatment, the combined treatment of 5 mmol·L^−1^ NH_4_Cl and 5 mmol·L^−1^ MSO decreased the proliferative effect compared to the single treatment of 5 mmol·L^−1^ NH_4_Cl (Fig. 4a). More MSCs were observed in the NH_4_Cl treatment group than in the combined treatment group under the microscope (Fig. 4b), and the cell number was decreased in the NH_4_Cl combined with 5 mmol·L^−1^ MSO treatment group compared to the NH_4_Cl treatment group via cell counting analysis (Fig. 4c). The results of the 7-week CCK-8 assays showed that the NH_4_Cl combined with MSO group had decreased cell proliferation compared to the single NH_4_Cl treatment group, and significant effects were observed on the 2nd day (Fig. 4d). Additionally, the percentage of S phase cells decreased when MSO was added, as shown by PI flow cytometry analysis (Fig. 4e, f). Subsequently, we detected the positive incorporation of BrdU following NH_4_Cl and MSO treatment in MSCs. Both the flow cytometry analysis and immunofluorescence analysis showed increased BrdU-positive cells in the single NH_4_Cl treatment group, while they decreased in the combined NH_4_Cl and MSO treatment group (Fig. 4g–j). These results further indicated that ammonia promotes MSC proliferation by conversion to glutamine via glutamine synthetase activity; furthermore, proliferation could be reduced by inhibiting glutamine synthetase.

### The different effects of ammonia on GS-expressing cells and cells with no expression of GS in bone marrow

**Fig. 5:**
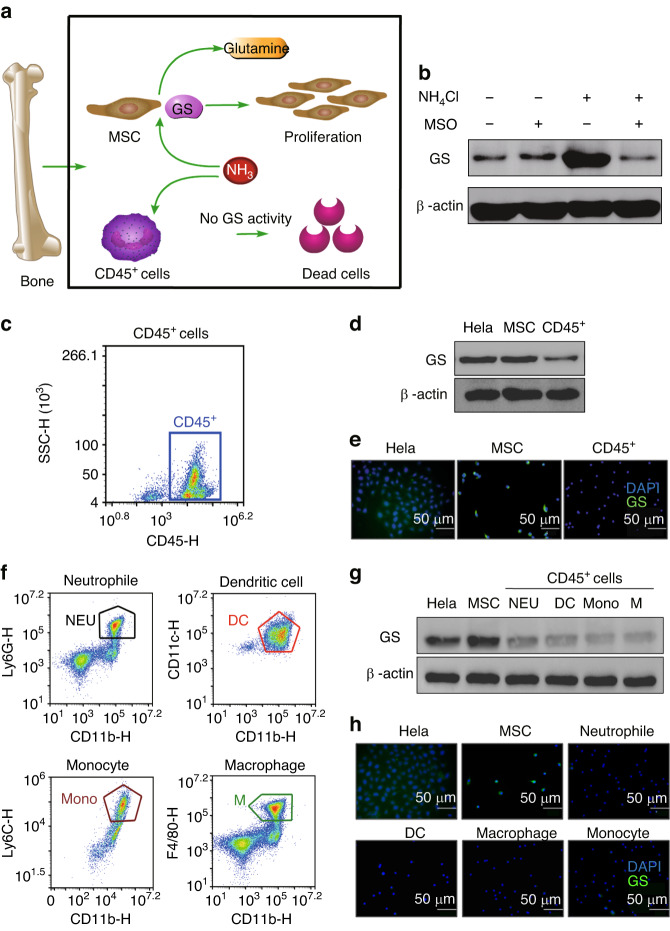
The different effects of ammonia on GS-expressing cells and cells with no expression of GS in bone marrow. **a** Schematic of the fates of ammonia in the GS-expressing cells or non-GS-expressing cells in bone marrow. **b** The expression of GS in MSCs with or without both NH_4_Cl treatment and MSO treatment via western blots. **c** CD45^+^ cells from mouse bone marrow sorted by flow cytometry. **d** The expression of GS analysis between MSCs and CD45^+^ cells via western blots, with HeLa cells as a positive control. **e** The expression of GS in MSCs and CD45^+^ cells via immunofluorescence, with HeLa cells as a positive control. **f** CD45^+^ cells, including neutrophils (*CD45*^*+*^ *CD11b*^*+*^ *Ly6G*^*+*^*)*, DCs *(CD45*^*+*^ *CD11b*^*+*^ *CD11c*^*+*^*)*, monocytes *(CD45*^*+*^ *CD11b*^*+*^ *Ly6C*^*+*^*)* and macrophages *(CD45*^*+*^ *CD11b*^*+*^ *F4/80*^*+*^*)*, were sorted by flow cytometry. **g** The expression of GS in MSCs, neutrophils, DCs, monocytes and macrophages via western blots, with HeLa cells as a positive control. **h** The expression of GS in MSCs, neutrophils, DCs, monocytes and macrophages via immunofluorescence. HeLa cells were used as a positive control

To determine the fate of ammonia in bone marrow, we further assessed the mechanism by which ammonia could promote MSC proliferation caused by the high expression of GS (Fig. 5b). CD45^+^ cells, such as neutrophils, DCs, monocytes and macrophages, without GS expression showed another fate: ammonia inhibited the growth of these cells as a toxic product. We sorted CD45^+^ cells from mouse tibias and femurs (Fig. 5c), and the expression of GS was reduced compared with that in MSCs (Fig. 5d, e). Furthermore, we sorted neutrophils, DCs, monocytes and macrophages via specific surface markers (Fig. 5f). The results showed that these cells notably reduced the expression of GS compared with MSCs (Fig. 5g, h). In summary, ammonia could promote proliferation in cells with GS expression, such as MSCs, but inhibited cells without GS expression, such as CD45^+^ cells in bone marrow (Fig. 5a).

### mTOR activation is involved in ammonia-induced mesenchymal stem cell proliferation

**Fig. 6:**
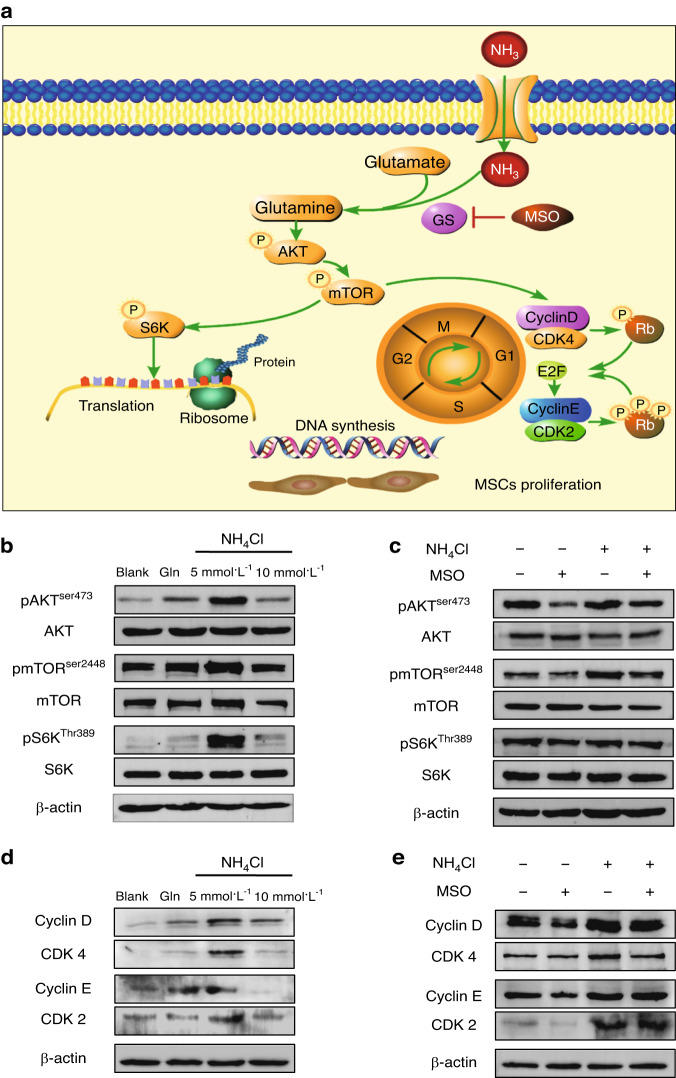
mTOR activation and cell cycle regulation are involved in ammonia-induced mesenchymal stem cell proliferation. **a** Schematic of the metabolic pathway of ammonia in GS-expressing MSCs. **b** Western blot analysis of the AKT/mTOR/S6K pathway with or without NH_4_Cl or glutamine treatment. **c** Western blot analysis of the AKT/mTOR/S6K pathway with or without both NH_4_Cl treatment or MSO treatment. **d** Western blot analyses of cyclin D/CDK4 and cyclin E/CDK2 with or without NH_4_Cl or glutamine treatment. **e** Western blot analysis of cyclin D/CDK4 and cyclin E/CDK2 with or without both NH_4_Cl treatment or MSO treatment

We explored the mechanism of MSC proliferation induced by ammonia. mTOR plays a fundamental role in supporting cell proliferation.^12^ As we mentioned previously, glutamine can activate the Akt/mTOR/S6K pathway by increasing phosphorylation of the mTOR pathway (Fig. 6a).^25^ Then, we investigated whether ammonia could activate the Akt/mTOR/S6K pathway by detecting phosphorylation of mTOR (Ser2448) and, upstream and downstream of mTOR, phosphorylation of Akt (Ser473) and S6K (Thr308) via western blot analysis.^26^ Our results showed enhanced phosphorylation of mTOR, Akt and S6K in the 5 mmol·L^−1^ NH_4_Cl treatment group, as well as the 5 mmol·L^−1^ glutamine treatment group, compared to the control group, and the 10 mmol·L^−1^ NH_4_Cl treatment group showed decreased phosphorylation of mTOR, Akt and S6K compared to the 5 mmol·L^−1^ NH_4_Cl treatment group, which is consistent with the proliferative effect of ammonia mentioned above. The expression of total mTOR, total Akt and total S6K showed no significant differences among the groups (Fig. 6b). We next assessed whether combined treatment with NH_4_Cl and MSO reduced the phosphorylation of mTOR (Ser2448), phosphorylation of Akt (Ser473) and phosphorylation of S6K (Thr308) compared to those of the NH_4_Cl treatment group. In addition, the expression of total mTOR, total Akt and total S6K showed no significant differences among the groups (Fig. 6c). These results revealed that ammonia could activate the Akt/mTOR/S6K pathway to promote MSC proliferation, and the activation of the Akt/mTOR/S6K pathway could be reduced by inhibiting the key enzyme for ammonia conversion to glutamine, glutamine synthetase, to decrease the effect of ammonia-induced proliferation on MSCs.

### Cell cycle regulatory proteins and DNA synthesis modulated by mTOR activation

We further investigated how ammonia promotes MSC proliferation regulated by the Akt/mTOR/S6K pathway. As mentioned above, we found an obvious increase in the percentage of S-phase cells in the NH_4_Cl treatment group (Fig. 2e) as well as in the glutamine treatment group (Fig. 3g), and this increase in S phase could be reduced by inhibition of glutamine synthetase (Fig. 4e). We further detected mTOR pathway-related cell cycle proteins and cyclin-dependent kinases, including cyclin D, CDK4, cyclin E and CDK2, which could induce cells from G1 phase to S phase to support MSC proliferation (Fig. 5a). The western blot results showed elevated expression of cyclin D, CDK4, cyclin E and CDK2 in the 5 mmol·L^−1^ NH_4_Cl treatment group (Fig. 6d). Elevated levels were also observed in the glutamine treatment group (Fig. 6d), and as expected, the expression of cell cycle proteins and cyclin-dependent kinases was reduced by inhibiting glutamine synthetase with combined treatment of NH_4_Cl and MSO compared to single treatment with NH_4_Cl (Fig. 6e). These results further revealed that activation of the Akt/mTOR/S6K pathway could regulate the distribution of the cell cycle to promote MSCs from G1 phase to S phase via activation of the cell cycle proteins and cyclin-dependent kinases related to proliferation.^27^ MSCs in S phase showed an increase in DNA synthesis for proliferation.

### The proportion of bone marrow-derived MSCs was elevated in ammonia-loaded mice

**Fig. 7:**
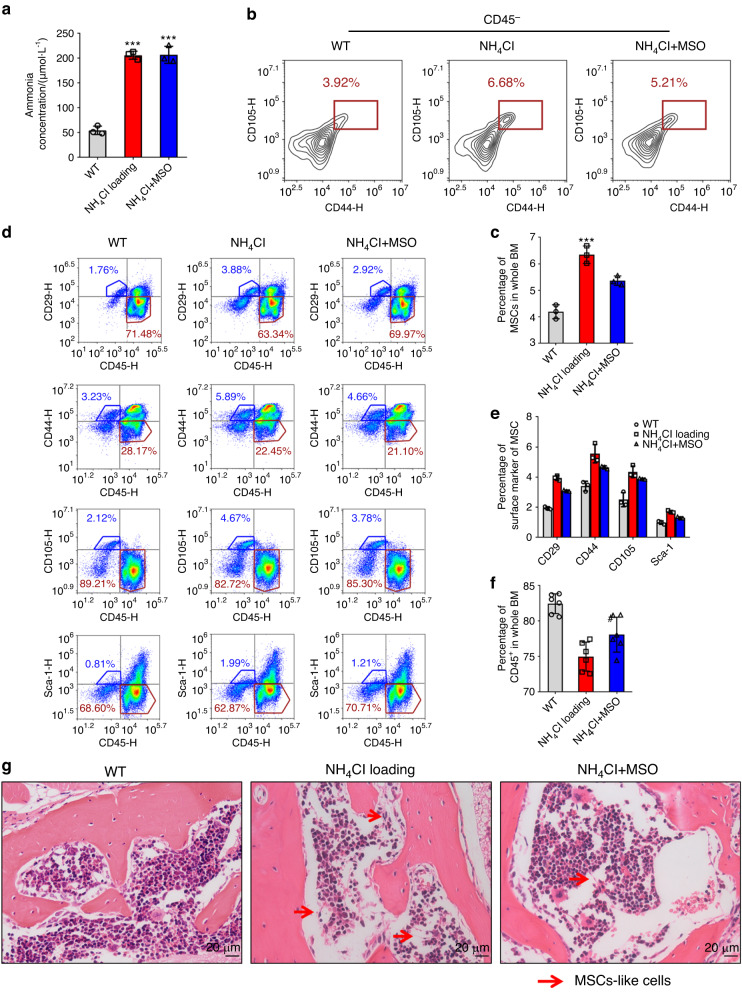
The proportion of bone marrow-derived MSCs was elevated in ammonia-loaded mice. **a** The ammonia concentration (µmol·L^−1^) of the wild-type mouse group, NH_4_Cl-loaded mouse group and NH_4_Cl-loaded mouse groups with MSO injection. **b** The percentage of MSCs in CD45- cells from mouse tibias and femurs in each group. **c** The statistical results of the percentage of MSCs in BM. **d** The percentage of the expression of MSC surface markers (CD29, CD44, CD105, Sca-1) and the expression of CD45 in each group. **e** The statistical results of the percentage of MSC surface marker expression in each group. **f** The statistical results of the percentage of CD45 expression in each group. **g** HE staining of the spinal column in each group. Red arrowheads, BM-derived fibroblasts; blue arrowheads, hematopoietic cells. Values are the mean ± SEM of an experiment performed in triplicate (one-way analysis of variance). *P < 0.05 versus the WT group. ^#^*P* < 0.05 versus the NH_4_Cl-loaded group

To investigate the growth conditions of bone marrow-derived MSCs with hyperammonemia in vivo, we established a chronic ammonia-loaded model. The ammonia level of the ammonia-loaded mice was significantly higher than that of the control group (Fig. 7a). To explore whether a high ammonia concentration in vivo could also promote MSC proliferation and whether proliferation could be inhibited by injection of MSO in vivo, we collected the tibias and femurs of the mice and digested them with collagenase, and then, the collected cells were analyzed by FACS. The percentage of ammonia-loaded mouse MSCs was elevated compared with that in the control group, and that in the ammonia-loaded mice with MSO injection was reduced (Fig. 7b, c). We further measured the proportion of CD45^+^ cells, which indicates the non-GS-expressing cells or MSCs from bone marrow. The ammonia-loaded mice showed an increase in MSCs and a decrease in CD45^+^ cells. (Fig. 7d–f). HE staining of the spinal column showed more fibroblasts and fewer hematopoietic cells in the ammonia-loaded mice than in the control mice (Fig. 7g). These results indicated that ammonia could not only promote MSC proliferation in vitro but also elevate the proportion of MSCs in vivo.

### The proportion of bone marrow-derived MSCs was elevated in the tumor infiltration model and uremic model

**Fig. 8:**
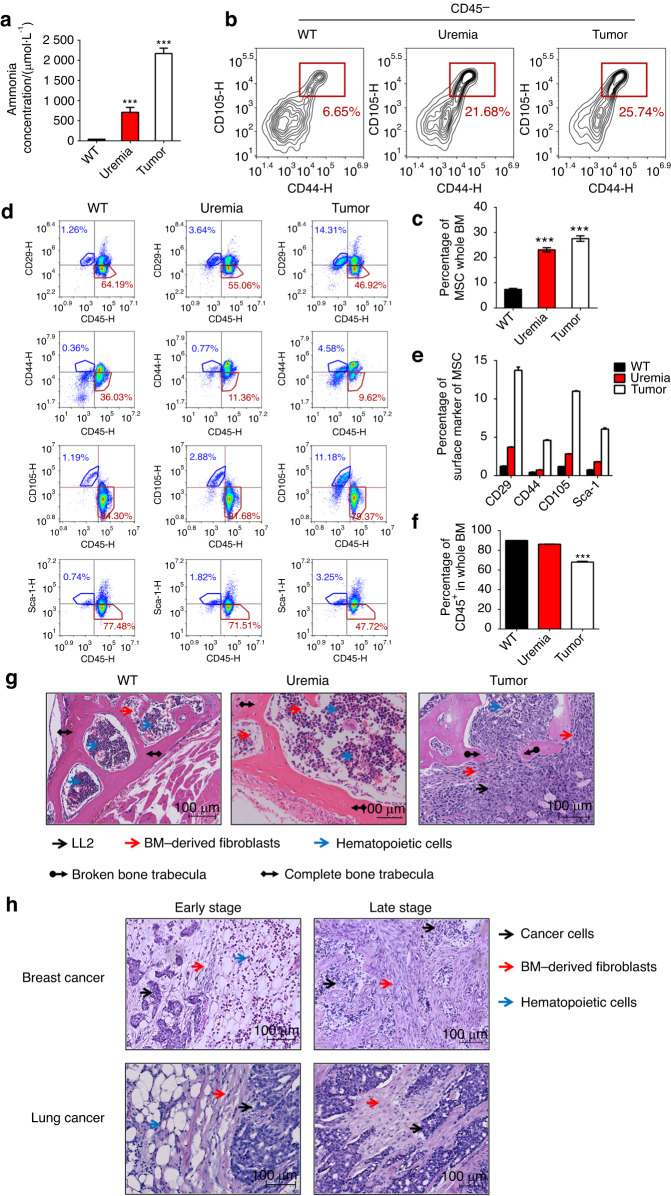
The proportion of bone marrow-derived MSCs was elevated in the tumor infiltration model and uremic model. **a** The ammonia concentration (µmol·L^−1^) of wild-type mice, uremia model mice and tumor infiltration mice. **b** The percentage of MSCs in CD45- cells from mouse tibias and femurs in each group. **c** The statistical results of the percentage of MSCs in BM. **d** The percentage of the expression of MSC surface markers (CD29, CD44, CD105, Sca-1) and the expression of CD45 in each group. **e** The statistical results of the percentage of MSC surface marker expression in each group. **f** The statistical results of CD45 expression among in group. **g** HE staining of the spinal column in each group. Red arrowheads, BM-derived fibroblasts; blue arrowheads, hematopoietic cells; black arrowheads, LL2 cells; black arrowheads with diamond, completed bone trabecula; black arrowheads with circle, broken bone trabecula. **h** HE staining of bone with infiltrating lung cancer or breast cancer cells from patients. Red arrowheads, BM-derived fibroblasts; blue arrowheads, hematopoietic cells; black arrowheads, cancer cells. Values are the mean ± SEM of an experiment performed in triplicate (one-way analysis of variance). *P < 0.05 versus the WT group

To further investigate the proportion of MSCs in bone tissue in vivo with both normal and high ammonia concentrations, we established two high-level ammonia concentration models: a uremic model and a tumor infiltration model.^28^ First, we detected the peripheral blood ammonia concentration of the untreated uremic model and tumor infiltration model. The results revealed an expected difference in the ammonia concentration of the tumor infiltration group and uremic group compared to the untreated group (Fig. 8a). To assess the distribution of MSCs and HSCs in bone tissues in vivo, we separated mouse femurs and tibias and assessed them by flow cytometry analysis. The results demonstrated that the positive markers of MSCs, including CD29, CD44, CD105 and Sca-1, increased in the uremic group and tumor infiltration group compared to the untreated group (Fig. 8b–e), while the positive markers of HSCs and CD45 decreased in the uremic group and tumor infiltration group compared to the untreated group (Fig. 8f). On the basis of these phenomena, to further assess the presence of MSCs in bone tissue in situ, we used spinal section staining with HE (Fig. 8g). The results revealed that more fibroblasts and fewer hematopoietic cells appeared at a higher proportion in both the uremic model group and the tumor model group compared to the untreated group. Moreover, we found that in the bone metastasis tissue sections of human lung cancer and breast cancer, more fibroblasts appeared around cancer cells, the residual hematopoietic cells appeared distal to the cancer cells at the early stage, and fibroblasts were present instead of myeloid cells around cancer cells at the late stage (Fig. 8h). These results showed that some pathological conditions with hyperammonemia, such as uremia or tumors, could also elevate the proportion of MSCs in vivo.

## Discussion

Ammonia is a ubiquitous byproduct of cellular metabolism. Under physiological conditions, the most important source of blood ammonia is deamination by amino acids.^8^ In addition, low levels of ammonia are absorbed by the intestine and secreted by the renal tubule epithelium. For the metabolism of ammonia, the synthesis of urea in the liver is the main pathway for blood ammonia.^29^ Additionally, the synthesis of glutamine and other nitrogen-containing compounds is an important pathway for ammonia metabolism. In balanced ammonia regulation, physiological concentrations of ammonia are maintained at 20 µmol·L^−1^ to 50 µmol·L^−1^. In pathological states of ammonia metabolic disorders, such as uremia or tumor conditions, the concentration of ammonia ranges from 1 mmol·L^−1^ to 5 mmol·L^−1^. When the balance of ammonia metabolism is disrupted, high levels of ammonia accumulation cause hyperammonia, which creates metabolic disorders,^29^ and ammonia in this situation is a toxic product.

As a toxic product, ammonia generally inhibits cell proliferation.^30^ However, after a series of related metabolic pathways, ammonia can be converted to nutritious amino acids. The most common nutritious product of metabolism is glutamine, which is generated via GS (glutamine synthetase). As mentioned previously, glutamine synthetase is a synthetic enzyme that catalyzes the synthesis of glutamine from glutamate and ammonia.^31^ When the concentration of ammonia in organisms is too high, glutamine is synthesized in large quantities via GS. This process not only provides nutritious amino acids for energy requirements but also protects against poisoning caused by excessive accumulation of ammonia. Our results revealed that ammonia could promote mesenchymal stem cell proliferation through the high levels of glutamine synthetase in MSCs instead of inhibiting their proliferation as a toxic substance. We also proved that MSCs could convert excess ammonia into glutamine by glutamine synthetase. MSO (methionine sulfoximine), a specific inhibitor of GS,^32^ reduced glutamine synthetase activity by converting ammonia to glutamine. After inhibition of glutamine synthetase by MSO, we found a reduction in the proliferative effect compared to that of the untreated group. Our results also demonstrated that as a nutritious amino acid, glutamine could activate the AKT/mTOR/S6K pathway, a classic nutritional pathway for cell proliferation,^33^ and mTOR could also regulate cell cycle-related proteins, which promote MSC transformation from the G1 phase to the S phase of the cell cycle, accompanied by an increase in DNA synthesis.^34^ In an in vivo study, we established a uremia model and tumor infiltration model as a high-level ammonia model. Our study also revealed that more MSCs were expressed in the high-level ammonia model than in the normal C57BL/6 mice.

Compared to previous research on ammonia, we proposed different fates for ammonia in bone marrow. Our results demonstrated that ammonia is no longer only a toxic product of metabolism in the traditional sense; it can also be used as a nutrient to promote cell proliferation. Our study revealed that the main mechanism that transforms toxic products into nutrients for proliferation is the activity of glutamine synthetase. Consequently, the fate of ammonia depends on the expression of glutamine synthetase in cells. In neutrophils, DCs, monocytes, or macrophages, no expression of GS was observed; in such a situation, ammonia, as a toxic product, does not promote GS-expressing cells in bone marrow dysfunction.^2,35^ This phenomenon also showed how the dysfunction of T cells or immunocytes in the tumor microenvironment might be caused by high levels of ammonia accumulation.^36^ Furthermore, ammonia promotes cells in the bone marrow with enhanced GS proliferative activity, such as MSCs^22^ with high expression of GS.

In summary, our study revealed that ammonia is no longer just a metabolic waste product;^37^ it can also be recycled to the nutritional amino acids required for cell proliferation.^38^ Although ammonia was previously considered to be a toxin, in our study, we demonstrated that ammonia can promote cell proliferation by converting ammonia into glutamine through GS activity.^39^ In addition, in the tumor microenvironment or other conditions with dysfunctional ammonia metabolism, ammonia accumulates and is utilized to synthesize amino acids by cancer cells or other cells with high expression of glutamine synthetase.^40^ These biosynthesis pathways support the systemic and autonomous metabolism of tumors and other GS-positive cells, such as the MSCs we mentioned above.^41^ Therefore, our study reveals that the metabolic cycle of ammonia provides an important nitrogen source for GS-expressing cells. For cells without expression of glutamine synthetase, such as neutrophils or DCs, ammonia could cause cell death as a toxic substance.

## Materials and methods

### Isolation and culture of bone marrow-derived mesenchymal stem cells

Mesenchymal stem cells were isolated from the femurs and tibias of 6-week-old female C57 mice from Vital River Laboratory Animal Technology (Beijing). Primary bone marrow-derived MSCs were cultured in DMEM (Dulbecco’s modified Eagle’s medium; Gibco) with 10% FBS (Gibco) and 1% penicillin/streptomycin. The nonadherent cells were removed after 24 h and changed to fresh medium. Then, fresh medium was added every 3 days, and the cells were passaged at a ratio of 1:2 when the MSCs reached approximately 80% confluence in culture dishes. MSCs at passages 4–6 were harvested for our experiment.

### Identification and differentiation of bone marrow-derived mesenchymal stem cells

For identification of the cells isolated from bone marrow, cell surface markers and multidifferentiation potential were analyzed. Cells at passages 4–6 were harvested and then resuspended in PBS containing 1 × 10^5^ cells. Cells were stained with MSC-related surface markers, including CD44-APC, Sca-1-FITC, CD29-FITC, and CD105-PE, and the negative markers of MSCs were CD31-PE, CD86-FITC, CD11b-FITC, CD34-PE, and CD45-APC (all from BD Pharmingen, Franklin Lakes, NJ, USA) for 30 min at 4 °C. The cells were resuspended in 200 µL of PBS for FACS analysis and incubated with isotype antibodies as a negative control. Furthermore, differentiation assays were performed in differentiation culture to prove that these cells can readily differentiate into osteoblasts by alkaline phosphatase staining, adipocytes by oil red O staining and chondrocytes by toluidine blue O staining.

### RT-PCR analysis

Total RNA was isolated from MSCs using TRIzol (Invitrogen/Thermo Fisher Scientific). Then, 1 μg of total RNA was used for reverse transcription. Next, the StepOnePlus Real-Time PCR System (ABI, Abilene, TX, USA) was prepared for quantitative real-time PCR. PCR was carried out in a volume of 20 μL using 10 pmol of each primer for MSC differentiation, 10 μL of Power SYBR Green PCR Master Mix (ABI, Valencia, CA, USA) and 5 μL of cDNA (5 ng·μL^−1^). The PCR was first performed with a 5 min preincubation period at 95 °C, followed by 40 cycles of 30 s each at 95 °C, 30 s at 56 °C, and 30 s at 72 °C. We performed all reactions in triplicate. mRNA expression was then normalized to that of glyceraldehyde-3-phosphate dehydrogenase (GAPDH). All primer sequences for qPCR are listed in Table 1.

**Table 1:** All primer sequences used for PCR

Primer	Forward (5ʹ-3ʹ)	Reverse (5ʹ-3ʹ)
Blgap	GGGCAATAAGGTAGTGAACAG	GCAGCACAGGTCCTAAATAGT
Runx2	TTACCTACACCCCGCCAGTC	TGCTGGTCTGGAAGGGTCC
Alpl	CACAATATCAAGGATATCGACGTGA	ACATCAGTTCTGTTCTTCGGGTACA
Acan	CACGCTACACCCTGGACTTTG	CCATCTCCTCAGCGAAGCAGT
Pparg	ACCACTCGCATTCCTTTGAC	TGGGTCAGCTCTTGTGAATG
GAPDH	AGGGCTGCTTTTAACTCTGGT	CCCCACTTGATTTTGGAGGGA

### Real-time cellular analysis

For determination of the proliferation curve of MSCs by the effect of ammonia within a week, RTCA (real-time cellular analysis) was performed. One x 10^4^ MSCs were plated in 96-well plates, and each concentration of NH_4_Cl or MSO had three replicates. We first cultured cells in plates for 8 h to ensure the adherence of MSCs before treatment with NH_4_Cl or MSO. Then, real-time detection was observed 7 days after each concentration of NH_4_Cl or MSO was added. Next, we analyzed the growth curve with time (days) as the horizontal axis and the cell index as the vertical axis to observe the effect of ammonia-induced proliferation of MSCs.

### Cell counting analysis

MSCs at passages 4–6 were harvested and then seeded in 24-well plates at a concentration of 1 × 10^4^ cells. We cultured these cells with or without treatment with ammonium chloride [NH_4_Cl; Sigma-Aldrich (Shanghai) Trading Company, Shanghai, China], glutamine (Sigma-Aldrich) and MSO (Sigma-Aldrich). The concentrations of these reagents are noted above. After 48 h of treatment, cells were harvested by removing the culture and adding 3 mL trypsin. After 3 min of digestion at room temperature, the collected cells were centrifuged for 3 min at 1 500 r·min^−1^ with 5 mL of fresh medium and then resuspended in 1 mL of fresh medium. The final cells were counted by a Countstar^®^ Automated Cell Counter (Shanghai, China) with a 10 µL cell suspension.

### Determination of glutamine

To assess the glutamine concentration of NH_4_Cl-treated MSCs, we seeded 1 × 10^6^ MSCs in 6-well plates and then cultured the cells with or without NH_4_Cl or MSO after 48 h. Then, the cells were harvested by removing the culture and adding 1 mL of trypsin. After 3 min of digestion at room temperature, the collected cells were centrifuged for 3 min at 1 500 r·min^−1^ with 5 mL of fresh medium and resuspended in ultrapure water for glutamine testing. The procedure for determining glutamine was based on the Glutamine/Glutamate Determination Kit (Sigma-Aldrich #GLN1).

### Cell proliferation assay

Cell proliferation was assessed by Cell Counting Kit-8 (CCK-8 Dojindo Laboratories #KJ798) and 5-bromodeoxyuridine proliferation assays. For the CCK-8 assay, we added 8 × 10^3^ MSCs in 96-well plates with or without NH_4_Cl and MSO treatment in triplicate. The CCK-8 assays were performed from 0 to 6 days after treatment with NH_4_Cl and MSO. Then, the medium was removed, and 100 μL of fresh medium in 10 μL of the CCK-8 solution was added for another 1 h at 37 °C. The OD at 450 nm was measured. The assay was repeated 3 times.

A 5-BrdU proliferation assay was used to measure the cell proliferative capacity. Passage 4–6 MSCs were seeded in 6-well plates at a concentration of 2 × 10^5^ cells per well after treatment with 48 h of NH_4_Cl or MSO. After incubation with BrdU (Sigma #B5002) for 2 h, the cells were fixed with methanol (−20 °C) for 20 min, acidized with 2 mol·L^−1^ HCl for 30 min at 37 °C and 0.1 mol·L^−1^ sodium borate for 15 min at room temperature and permeabilized with 0.5% Triton X-100 for 20 min. Then, anti-5-BrdU antibody (Cell Signaling Technology cat #5292) was added overnight at 4 °C. Finally, we detected the incorporation rate of BrdU via flow cytometry analysis and immunofluorescence analysis.

### Cell cycle analysis

Cell cycle analysis was performed with PI staining and flow cytometry. We seeded MSCs in 6-well plates at a concentration of 2 × 10^5^ cells per well. After 48 h of treatment with NH_4_Cl or MSO, the cells were harvested, fixed with ethanol (−20 °C) overnight at 4 °C and then measured via flow cytometric analysis after PI staining for 15 min at 4 °C. The proportion of cells in each cell cycle (G1 phase, S phase, and G2 phase) was measured by ACEA Novoexpress software.

### Western blot analysis

MSCs at passages 4–6 were harvested and seeded in 6-well plates at a density of 1 × 10^6^ cells per well after 48 h of treatment with NH_4_Cl or MSO. The treated cells were then harvested in 100 µL of RIPA lysis buffer (Beyotime, China) with 0.5% protease inhibitor cocktail for 30 min at 4 °C, and the samples were centrifuged at 13 000 r·min^−1^ for 15 min at 4 °C. For determination of the protein concentration, a BCA protein assay kit (Thermo Scientific, USA #23225) was used. Then, equal amounts of protein samples were electrophoresed on a 7.5% gradient gel and transferred to a PVDF (polyvinylidene fluoride) membrane. The membranes were then blocked with 5% milk in TBST (Tris-buffered saline with 0.1% Tween-20) at room temperature for 2 h and incubated with specific primary antibodies overnight at 4 °C. The primary antibodies included those against the AKT/mTOR/S6K pathway proteins, such as total-AKT Cell Signaling Technology #4685, p-AKT (Ser473) (Cell Signaling Technology #4060), total mTOR (Cell Signaling Technology #2983), p-mTOR (Ser2448) (Cell Signaling Technology #5536), total-S6K (Cell Signaling Technology #2708), and p-S6K (Thr389) (Cell Signaling Technology #97596), and the cell cycle-related proteins, including cyclin D1 (Cell Signaling Technology #2978), CDK4 (Cell Signaling Technology #12970), cyclin E1 (Cell Signaling Technology #20808) and CDK2 (Cell Signaling Technology #2546). We used β-actin (Cell Signaling Technology #3700) as a reference. These blots were washed with TBST 3–5 times and incubated with a horseradish peroxidase (HRP)-conjugated secondary antibody (Santa Cruz Biotechnology) for 90 min at room temperature. The final blots were measured by the chemiluminescence method via a Bio-Rad ChemiDoc Imaging System.

### Ammonia-loading experiments

C57 mice were fed 0.28 mol·L^−1^ NH_4_Cl solution to replace water, and the mice in the MSO treatment group were injected with MSO (50 mg·kg^−1^ i.p.). After 2 weeks, the treated mice were sacrificed, and peripheral blood was collected. The ammonia concentration in the blood was measured using an Ammonia Assay Kit [Sigma-Aldrich (Shanghai) Trading Company]. The tibia and femur of both the wild-type group and the treated group were collected for flow analysis and immunohistochemical analysis to measure the distribution of MSCs in bone marrow.

### Expression of GS activity analysis

Primary bone marrow cells from the femurs and tibias of C57 mice were collected and sorted by flow cytometry. The MSCs were cultured according to the steps described above. HeLa cells were used as a positive control of GS expression. GS activity was measured by western blot and immunofluorescence analysis with anti-glutamine synthetase (ab178422 Abcam China).

### Surgical procedure for uremia

Our experiments were performed in 8-week-old female C57BL/6 mice. The renal failure surgical procedure involved two steps. First, we exposed the right kidney through a minimal incision. Then, we applied electrocautery to the kidney cortex over the entire surface of the right kidney, while part of the hilum and a small portion of the upper region remained intact. Then, the right kidney was gently pushed back into the retroperitoneal cavity. Second, 2 weeks after the first operation, the total left kidney was removed through a similar incision as that on the right side. Then, the left renal vascular pedicle and ureter were carefully tied. We completely stitched up the incision. In the control group animals, the right kidney underwent electrocautery, and the left kidney was exposed but not removed and completely stitched up in the sham operation. All mice were studied 12 weeks after the second operation.

### Tumor infiltration model experiments

Tumor infiltration experiments were performed in 4- to 6-week-old female C57BL/6 mice. Each C57 mouse in the experimental group was injected next to the spinal column with LL2 cells at a concentration of 2 × 10^6^ and injected with normal saline as a control. Mice were sacrificed after 2 weeks, the spinal columns of each group were collected for the analysis of HE staining, and the femurs and tibias of each group were collected for flow cytometry analysis.

### Statistical analysis

Comparisons between different conditions were analyzed with a two-tailed, paired Student’s *t* test (T test) or with one-way analysis of variance (ANOVA). *P* < 0.05 was considered statistically significant.
