# Author Correction: Ammonia promotes the proliferation of bone marrow-derived mesenchymal stem cells by regulating the Akt/mTOR/S6k pathway

**DOI:** 10.1038/s41413-024-00314-y

**Published:** 2024-02-20

**Authors:** Yu Liu, Xiangxian Zhang, Wei Wang, Ting Liu, Jun Ren, Siyuan Chen, Tianqi Lu, Yan Tie, Xia Yuan, Fei Mo, Jingyun Yang, Yuquan Wei, Xiawei Wei

**Affiliations:** 1 Laboratory of Aging Research and Cancer Drug Target, State Key Laboratory of Biotherapy and Cancer Center, National Clinical Research Center for Geriatrics, West China Hospital, Sichuan University, No. 17, Block 3, Southern Renmin Road, Chengdu, Sichuan 610041 PR China; 2 Department of Clinical Laboratory, The West China Second University Hospital of Sichuan University (WCSUH-SCU), Sichuan University, No. 17, Block 3, Southern Renmin Road, Chengdu, Sichuan 610041 PR China; 3 Department of Prenatal Diagnosis Center, The West China Second University Hospital of Sichuan University (WCSUH-SCU), Sichuan University, No. 17, Block 3, Southern Renmin Road, Chengdu, Sichuan 610041 PR China

**Keywords:** Bone cancer, Bone

Correction to: *Bone Res*earch 10.1038/s41413-022-00215-y, published online 26 August 2022

After online publication of the article, the authors identified inadvertent mistakes occurred in Fig. 1 that requires correction. In Figure 1c two micrographs have been duplicated and presented with different experimental conditions. The revisions cause some changes but do not affect any conclusions of the current work and the description of the whole article.

The correct Figure 1c and the accompanying legend appear below:
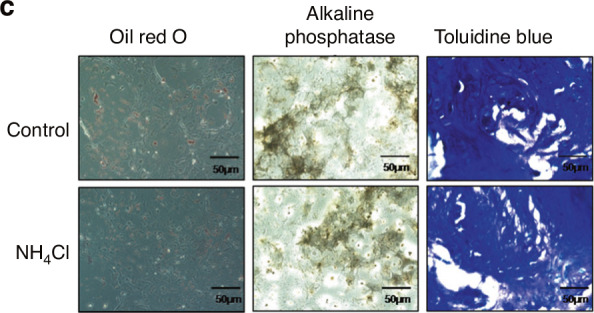


Fig. 1**c**. Isolation and identification of mesenchymal stem cells from mouse bone marrow. c. Adipogenesis of MSCs was observed with Oil red O staining, osteoblastogenesis was assayed with in situ alkaline phosphatase staining, and chondrocytic cells were identified with toluidine blue staining.

After online publication of the article, the authors identified inadvertent mistakes occurred in Fig. 5 that requires correction. The original version of this article contains an error in Figure 5d and 6c, a western immunoblot has been duplicated and presented with different experimental conditions. The revisions cause some changes but do not affect any conclusions of the current work and the description of the whole article.

The correct Figure 5d and the accompanying legend appear below:
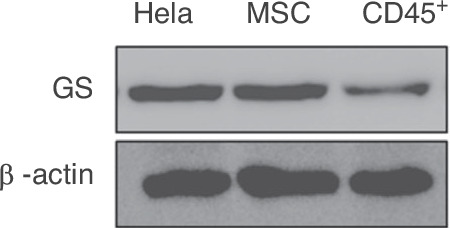


Fig. 5**d**. The different effects of ammonia on GS-expressing cells and cells with no expression of GS in bone marrow. d. The expression of GS analysis between MSCs and CD45^+^ cells via western blot, with HeLa cells as a positive control.

The original article^1^ has been updated.
